# Descendants of hypertrophic chondrocytes promote angiogenesis by secreting THBS4 during bone growth and injury repair

**DOI:** 10.1038/s41413-025-00469-2

**Published:** 2025-11-10

**Authors:** Shiju Song, Jing Fan, Guangyu Ding, Jinhua Yin, Weiguang Lu, Liangjie Huang, Jingyan Hu, Xueqin Gong, Bo Gao, Qiang Jie, Kathryn Song Eng Cheah, Chao Zheng, Liu Yang

**Affiliations:** 1 Institute of Orthopedic Surgery, Xijing Hospital, Fourth Military Medical University, Xi’an, China; 2 Institute of Medical Research, Northwestern Polytechnical University, Xi’an, China; 3 School of Medicine, Northwest University, Xi’an, China; 4 Pediatric Hospital, Honghui Hospital, Xi’an Jiaotong University, Xi’an, China; 5 Xi’an Key Laboratory of Skeletal Developmental Deformity and Injury Repair, Xi’an, China; 6 Research Center for Skeletal Developmental Deformity and Injury Repair, School of Life Science and Medicine, Northwest University, Xi’an, China; 7 School of Biomedical Sciences, University of Hong Kong, Hong Kong, China; 8 Innovation Research Institute, Xijing Hospital, Fourth Military Medical University, Xi’an, China

**Keywords:** Bone, Bone quality and biomechanics

## Abstract

Hypertrophic chondrocytes (HCs) could transform into osteoblastic lineage cells while the pathophysiological implications of HC transformation remain largely unknown. Here, we generated a mouse line utilizing *Col10a1-Cre* to induce DTA expression to genetically ablate HCs and their descendants. *Col10a1-Cre; R26*^*DTA/+*^ mice displayed dwarf phenotype, abnormal spongy bone, and significantly delayed drill-hole injuries healing, suggesting an indispensable role of HC lineage extension in bone growth and injury repair. Intriguingly, single-cell RNA sequencing analysis revealed the most significant loss of a cell cluster expressing multiple angiogenic factors (Pro-Angiogenic Descendants of HCs, PADs) among cells derived from *Col10a1-Cre; R26*^*DTA/+*^ and control femurs. In silico analysis of cell-cell communication supported Thrombospondin 4 (THBS4) as a specific angiogenic factor mediating the crosstalk between PADs and vascular endothelial cells. Concordantly, analyses using immunostaining combined with tissue clearing revealed that PADs physically contacted with endothelial cells, whereas *Col10a1-Cre; R26*^*DTA/+*^ mice showed defective metaphyseal and cortical vessel formation and post-injury angiogenesis along with a significant loss of THBS4. Moreover, in vitro assays showed that supplying THBS4 was sufficient to promote proliferation and tube formation of endothelial cells and rescue defective angiogenesis of *Col10a1-Cre; R26*^*DTA/+*^ metatarsal explants. Collectively, these findings demonstrate a critical role of PADs in bone growth and injury repair by secreting THBS4 to regulate angiogenesis.

## Introduction

The appendicular skeleton forms primarily via endochondral ossification.^1,2^ During the early endochondral bone development after birth, it has been found that osteoblasts derived from hypertrophic chondrocytes (HCs) contributed to approximately 60% of the osteoblasts.^3^ Osteoblasts originating from HCs predominantly populated the metaphyseal endosteum, cortical bone, trabeculae, and marrow, being scarce or absent in the diaphysis, and contributed to bone formation from embryogenesis through early postnatal life, remaining visible in mice at eight months of age.^3–6^ By constructing mice with specific gene knockouts in HCs, previous studies revealed that genes such as *Ctnnb1*, *Irx3*, *Irx5*, *Runx2*, and *Ptch1* were key regulators of the cell fate of HC descendants.^7–10^ In addition, MMP14 was reported to control the differentiation and survival of HC-derived osteoblasts by modulating the PTH/PTH1R pathway.^11^ Recent single-cell sequencing studies further revealed that HCs could transform into skeletal stem cells, suggesting HCs can contribute to the reservoir of skeletal stem cells.^12,13^ Skeletal stem cells derived from the transformation of HCs, along with Dlx5^+^ cells in the perichondrium, Lepr^+^ and Fgfr3^+^ cells in the marrow cavity, and Gli1^+^ cells in the periosteum, jointly participated in the development and injuries repair of long bones.^14–19^ Despite these advances focusing on osteochondral lineage cells, whether and how HC transformation impacts other indispensable elements during bone formation, e.g., vasculature, remains to be elucidated.

It has been well established that osteogenesis is coupled with angiogenesis.^20–22^ Researches have indicated that proteinases like MMP13 and growth factors such as VEGF released by terminal HCs were crucial for the ingrowth of blood vessels into HC matrix.^21,23,24^ Moreover, in the same year when HCs transition to osteoblasts was identified, Type H capillaries were found in the metaphysis and endosteum and proved to mediate neo-angiogenesis in bone.^25^ Studies indicated that Type H capillaries-mediated cartilage replacement guided bone elongation and that disrupting the alignment of angiogenic blood vessels led to deformed bone shapes.^26^ Notably, a recent study reported that, in addition to HCs, descendants of HCs also positioned near blood vessels in bone marrow, and colony-forming units derived from HCs enriched expression of genes involved in vascular development.^12^ However, it is unclear whether and how the descendants of HCs regulate angiogenesis.

Herein, we set out to elucidate the pathophysiological implications of HC transformation during bone development and injury repair, as well as to decipher crosstalk between HC descendants and vascular endothelial cells. Utilizing a genetic lineage ablation model, we report that *Col10a1-Cre; R26*^*DTA/+*^ mice displayed a dwarfism phenotype, impaired trabecular bone structure, and prolonged healing of drill-hole injuries, underscoring an essential role of HC lineage extension in bone development and repair. Single-cell sequencing was employed to identify a unique subset of HC descendants involved in angiogenesis, and their close association with endothelial cells was then verified using fluorescence staining and tissue optical clearing methods. In vitro functional assays supported Thrombospondin 4 (THBS4) as a specific factor mediating the pro-angiogenic effects of HC descendants. Together, our study indicates direct implications of HC lineage extension in bone growth and injury repair by secreting THBS4 to regulate angiogenesis.

## Results

### Depletion of HCs and their descendants impaired endochondral ossification

**Fig. 1:**
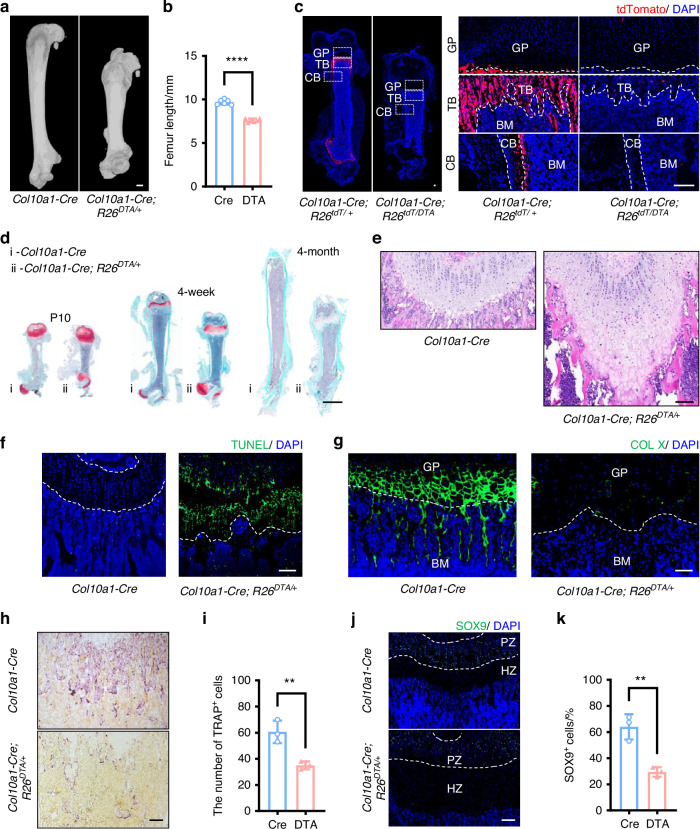
HCs-specific DTA expression led to severe limb abnormalities with impaired endochondral ossification. **a** Representative 3D reconstruction images of femurs from 4-week mice. **b** Statistical analysis of femur length (*n* = 6) in (**a**). **c** Representative images of tdTomato expression at low magnification (left) and high magnification (right) from P10 mice. **d** Representative images of Safranin O/Fast Green staining from P10, 4-week, and 4-month mice. **e** Representative images of HE from 4-week mice. **f** Representative images of IF staining for TUNEL from 4-week mice. **g** Representative images of IF staining for COL Ⅹ from P10 mice. **h** Representative images of TRAP staining from P21 mice. **i** Statistical analysis of TRAP^+^ cells (*n* = 3) in (**h**). **j** Representative images of IF staining for SOX9 from P10 mice. **k** Statistical analysis of SOX9^+^ cells (*n* = 3) in (**j**). The scale bar in (**a**) is 500 μm, in (**c**), (**e**), (**f**), (**g**), (**h**), and (**j**) is 100 μm, and in (**d**) is 2 mm. GP growth plate, TB trabecular bone, CB cortical bone, BM bone marrow, PZ proliferative zone, HZ hypertrophic zone

To elucidate the pathophysiological significance of cells derived from HCs in skeletal formation, we generated a novel mouse line by crossing *Col10a1-Cre*^27^ with *ROSA-DTA*^28^ mice. From postnatal day 10 (P10) on, *Col10a1-Cre; R26*^*DTA/+*^ mice began to show shorter limbs and dwarfism. The differences in stature and weight became pronounced at the age of four weeks (Fig. S1a, b), and *Col10a1-Cre; R26*^*DTA/+*^ mice showed a shorter lifespan than controls (Fig. S1c). Analysis using micro-computed tomography (micro-CT) indicated that the *Col10a1-Cre; R26*^*DTA/+*^ mice displayed impaired skeletal development, including decreased body length, globular skulls, smaller ribs, and defective spinal development (Fig. S1d). Specifically, the mutant mice displayed a femur length reduction of approximately 2.0 mm, about 20.0% shorter compared to the control mice (Fig. 1a, b). To confirm the efficiency of DTA-mediated cell linage ablation, we constructed *Col10a1-Cre; R26*^*tdT/+*^ and *Col10a1-Cre; R26*^*tdT/DTA*^ mice. In agreement with previous findings tdTomato signals were detected in the hypertrophic zone (HZ) and extended to the trabecular bone, endosteum, cortical bone, and bone marrow cavity of *Col10a1-Cre; R26*^*tdT/+*^ mice.^3–5^ *Col10a1-Cre; R26*^*tdT/DTA*^ mice showed nearly undetectable tdTomato signals, suggesting the potent efficacy of DTA in ablating cells derived from HCs (Fig. 1c). Safranin O/Fast Green staining demonstrated increasingly severe shortened deformities in *Col10a1-Cre; R26*^*DTA/+*^ mice from P10 to the age of 4 months, characterized by delayed secondary ossification, growth plate thickening, and reduced, distorted trabeculae (Fig. 1d). *Col10a1-Cre; R26*^*DTA/+*^ mice showed an expanded hypertrophic zone (HZ) (Fig. 1e). Notably, TUNEL staining revealed massive TUNEL-positive HCs and prominent matrix staining in *Col10a1-Cre; R26*^*DTA/+*^ mice, whereas TUNEL-positive cells were scarce in the control HZ (Fig. 1f). Moreover, type X collagen (COLX) was undetected in the expanded HZ of *Col10a1-Cre; R26*^*DTA/+*^ mice (Fig. 1g), and the TRAP-positive osteoclast count and staining intensity were markedly decreased in *Col10a1-Cre; R26*^*DTA/+*^ mice (Fig. 1h, i). These findings suggest that the accumulation of numerous dead HCs and loss of matrix-degrading osteoclasts might jointly cause the expansion of the HZ. Additionally, we observed a marked decrease in the population of SOX9^+^ chondrocytes within the proliferating zone in *Col10a1-Cre; R26*^*DTA/+*^ mice, suggesting that the proliferation of chondrocytes was compromised also (Fig. 1j, k).

### *Col10a1-Cre; R26*^*DTA/+*^ mice showed defective bone growth and injury repair

**Fig. 2:**
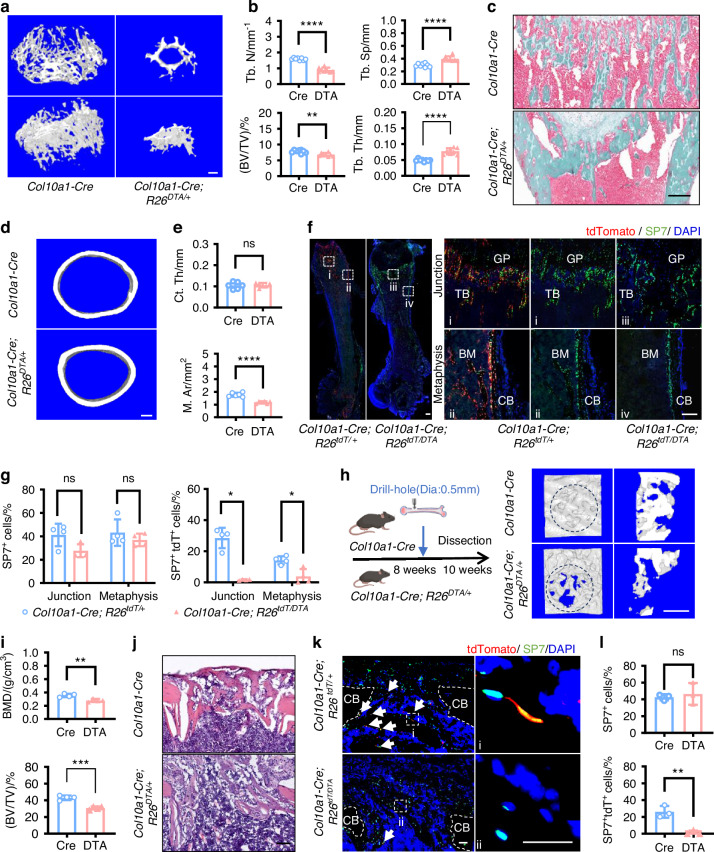
HCs deletion compromised trabecular bone formation and delayed bone repair. **a** Representative 3D reconstruction images of TB from 4-week mice. **b** Statistical analysis of trabecular parameters (*n* = 8) in (**a**). **c** Representative images of Goldner staining from 4-week mice. **d** Representative images of CB. **e** Statistical analysis of cortical parameters (*n* = 6) in (**d**). **f** Representative images of IF staining for SP7 at low magnification (left) and high magnification (right) from 4-week mice. **g** Statistical analysis of SP7^+^ and SP7^+^ tdTomato^+^ cells (*n* = 4) in (**f**). **h** Timeline (left) and 3D reconstruction images (right) from drill-hole injury experiments. **i** Statistical analysis of the drill-hole parameters (*n* = 4) in (**h**). Representative images of HE **j** and SP7 staining **k** from drill-hole injury experiments. White arrows: SP7^+^ tdTomato^+^ cells. **l** Statistical analysis of SP7^+^ and SP7^+^ tdTomato^+^ cells (*n* = 3) in (**k**). The scale bar in (**a**), (**d**), and (**h**) is 250 μm; and in (**c**), (**f**), (**j**), and (**k**) is 100 μm. GP growth plate, TB trabecular bone, CB cortical bone, BM bone marrow, tdT tdTomato

To investigate the alterations in bone formation, we performed micro-CT analysis and found in *Col10a1-Cre; R26*^*DTA/+*^ mice that the number and volume of trabeculae were reduced, leading to an increase in the spacing, whereas the thickness of the trabecular bone showed a notable increase of about 60% (Fig. 2a, b, Fig. S2a). Goldner-stained frozen sections of mouse femurs demonstrated disrupted trabecular organization in *Col10a1-Cre; R26*^*DTA/+*^ mice (Fig. 2c). The cortical bone thickness remained comparable between *Col10a1-Cre; R26*^*DTA/+*^ and control mice, while the marrow cavity of *Col10a1-Cre; R26*^*DTA/+*^ mice was significantly reduced in size (Fig. 2d, e). Despite structural and length alterations in the femurs of *Col10a1-Cre; R26*^*DTA/+*^ mice, three-point bending test revealed only a non-significant decrease in their bones’ maximum load and elastic modulus (Fig. S2b). At the cellular level, we noted that SP7^+^ osteoblasts primarily resided in the osteochondral junction and metaphysis. The average percentage of these cells in two regions was comparable between *Col10a1-Cre; R26*^*DTA/+*^ mice and controls, with a slight reduction in the average percentage of SP7^+^ osteoblasts at the osteochondral junction in *Col10a1-Cre; R26*^*DTA/+*^ mice, though the difference was insignificant. *Col10a1-Cre; R26*^*DTA/+*^ mice showed a marked decline in osteoblasts derived from HCs (SP7^+^ tdT^+^) at the osteochondral junction and metaphysis (Fig. 2f, g). Using a drilling injury model, we set out to investigate whether HC transformation contributes to injury repair. Two weeks post-injury, micro-CT revealed a significant reduction in callus bone mineral density and mass in *Col10a1-Cre; R26*^*DTA/+*^ mice, compared to controls (Fig. 2h, i). In line with this, HE-stained frozen tissue sections showed that *Col10a1-Cre; R26*^*DTA/+*^ mice had an abundance of cartilaginous callus at the injury site. While the number of SP7-stained osteoblasts in the callus was unchanged, there was a notable decrease in osteoblasts from the HCs (SP7^+^ tdT^+^) in *Col10a1-Cre; R26*^*DTA/+*^ mice (Fig. 2j–l). The above results suggested that alternative sources of osteoblasts might incompletely compensate for the deficiency of osteoblasts derived from HCs in bone growth and injury repair, suggesting an indispensable role of HCs and their descendants.

### Single-cell RNA sequencing revealed a pro-angiogenic subpopulation of HC descendants

**Fig. 3:**
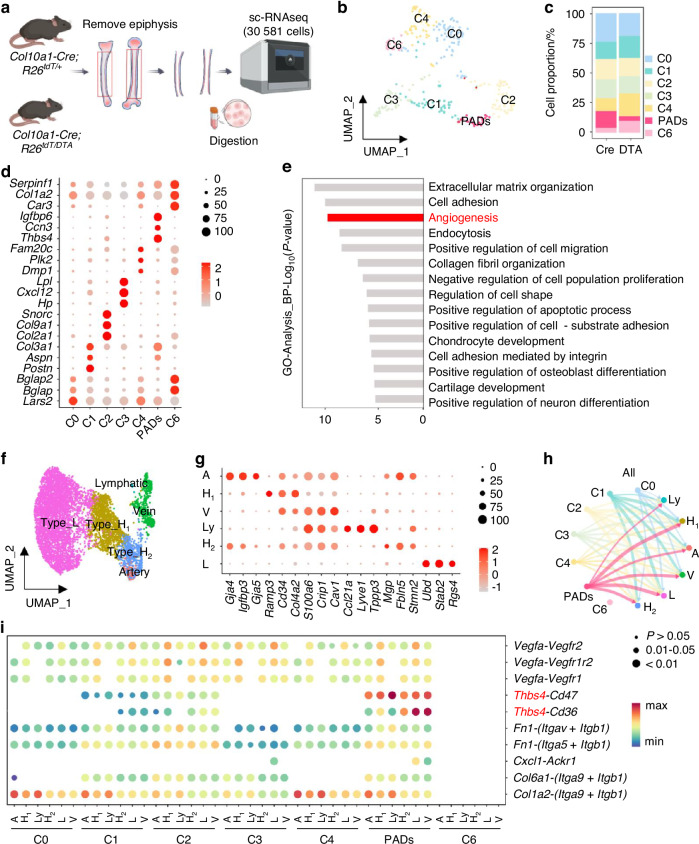
Single-cell sequence suggested that HCs’ descendants could promote angiogenesis. **a** Diagram of the single-cell datasets isolated from 4-week mice. **b** UMAP visualization of major sub-clusters of chondro-osteogenic cells. **c** Bar plots depicting the proportion of subclusters in indicated groups of mice. **d** Dot plot depicting the expression of top 3 marker genes of each cluster in chondro-osteogenic cells. Dot color represented gene expression levels, while dot size matched the proportion of cells expressing the gene. **e** GO enrichment analysis of biological processes for PADs marker genes. **f** UMAP visualization of major sub-clusters of endothelial cells. **g** Dot plot showed top 3 marker genes expression in endothelial cell clusters, with color for expression levels and size for cell expression percentage. **h** CellChat interaction analysis of chondro-osteogenic cells from *Col10a1-Cre; R26*^*tdT*/+^ mice and endothelial cells. **i** Bubble plot showed ligand-receptor interactions between these cells. Bubble size indicated significance, while color represented communication potential

To investigate HC descendants at single-cell level, we harvested femurs from 4-week-old *Col10a1-Cre; R26*^*tdT/+*^ and *Col10a1-Cre; R26*^*tdT/DTA*^ mice. To enrich HC descendants embedded in the metaphyseal endosteum and cortical bone, we removed the epiphyses and flushed out the bone marrow to avoid interference from HCs in the growth plate and trabecular bone, and marrow-resident hematopoietic cells. Finally, we captured and sequenced the transcripts of 30 581 cells located in the endosteal and cortical bone compartments (Fig. 3a). After dimensionality reduction and clustering, we manually annotated nine cell clusters (Fig. S3a).^29^ Among the annotated cell clusters, eight were related to hematopoiesis, and one distinct cluster specifically expressed early chondrogenic markers (*Sox9*),^30,31^ osteogenic(*Bglap2*)^32^ as well as genes related to skeletal stem cells (*Lepr/Cxcl12*),^17,19,33^ characterizing it as a chondro-osteogenic cell type (Fig. S3b). Further analysis of the chondro-osteogenic cells enabled their classification into seven unique subtypes^33,34^ (Fig. 3b). The C3 subset was enriched for genes associated with stem cell characteristics (*Lepr*/*Cxcl12*/*Adipoq*).^33,35^ Subsets C0, C4, and C6 highly expressed osteogenic genes (*Bglap*/*Dmp1/Col1a2*).^32,36,37^ The C2 subset highly expressed chondrogenic genes (*Col2a1*/*Col9a1*/*Sox9*).^30,31,38–40^ The C1 subset highly expressed specific genes of the periosteum (*Postn*/*Col3a1/Aspn)*^17,41,42^ (Fig. S3c). Furthermore, the cellular proportion analysis revealed a significant decrease in a specific cell cluster (C5) in *Col10a1-Cre; R26*^*tdT/DTA*^ mice, implying the most affected cell cluster by DTA ablation (Fig. 3c). Notably, the most affected cell cluster by DTA ablation, C5, was enriched for genes linked to angiogenesis promotion, like *Thbs4*^43,44^ and *Ccn3*^45^ (Fig. S3c, Fig. 3d, e). We herein designated these pro-angiogenic descendants of HCs as PADs. To elucidate the specific ligand-receptor pairs mediating communication between HC-derived cells and endothelial cells, we re-analyzed single-cell sequencing data from studies with age-matched endothelial cells.^46^ Endothelial cells from ten clusters were reclassified into six unique populations (Fig. 3f), as follows: *Gja5*^+^ *Sema3g*^+^ *Bmx*^+^ arterial cells(A), *Ramp3*^+^ *Aplnr*^+^ *Lamb1*^+^ Type H_1_ cells(H_1_), *Stab2*^+^ *Vcam1*^+^ *Rgs4*^+^ Type L cells(L), *Ramp3*^-^ *Efnb2*^+^ *Sox17*^+^ Type H_2_ cells(H_2_), *Lyve1*^+^ *Pdpn*^+^ *Prox1*^+^ lymphatic cells(Ly), and *Emcn*^+^ *Vwf*^+^ venous cells(V) (Fig. S3d).^43,44,47,48^ We identified that H-type cells could be subdivided into two subtypes based on the expression of *Ramp3*. The *Ramp3*^+^ Type H_1_ cells resembled Type L, whereas Type H_2_ aligned more closely with arterial cells (Fig. 3g). CellChat analysis revealed that PADs exhibited strong potential for interacting with endothelial cells compared to other chondro-osteogenic cells (Fig. 3h).^49^ We pinpointed factors such as *Vegfa, Thbs4*, *Fn1*, *Cxcl1*, *Col6a1*, and *Col1a2*, secreted by PADs to signal endothelial cells (Fig. 3i). This uncovered the intricate and dynamic interactions between PADs and endothelial cells, which were largely absent in *Col10a1-Cre; R26*^*tdT/DTA*^ mice. Our further results indicated that PADs likely communicated with endothelial cells through the *Thbs4*-(*Cd36*/*Cd47*) pathway and exhibited a significant potential for interaction with endothelial cells compared to other chondro-osteogenic cells via THBS signaling (Fig. 3i, Fig. S4a). PADs had the highest *Thbs4* expression among other populations (Fig. S4b) and *Cd47* was broadly expressed in endothelial cells, whereas *Cd36* expression was mainly found in lymphatic, Type L, and vein cells (Fig. S4c), suggesting THBS4 might act on endothelial cells mainly through CD47. To preliminarily validate the applicability of these findings to human, we projected mouse PADs to a published single-cell atlas of human skeletal stem cells^50^ and found most of the PADs were mapped onto human osteo-chondrogenic progenitor (OCP), where THBS4 was mainly expressed (Fig. S4d-g).

### Depletion of PADs led to defective metaphyseal and cortical vessel formation and post-injury angiogenesis

**Fig. 4:**
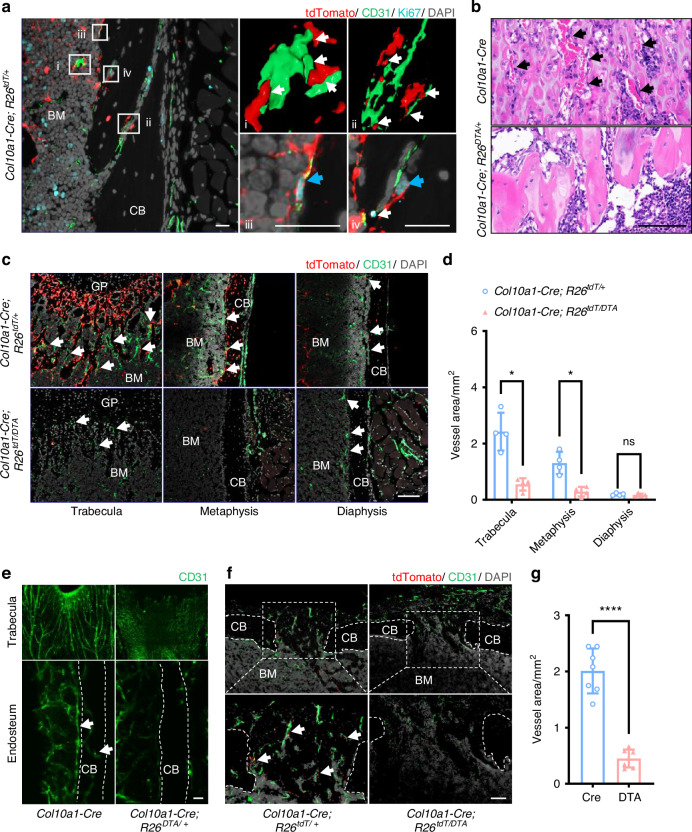
Loss of descendants of HCs reduced angiogenesis. **a** Representative IF images showing co-staining for CD31 and Ki67 in 4-week mice. (i-iv) Higher-magnification views. White arrows: contacts between tdTomato^+^ cells and endothelial cells; Blue arrows: proliferating endothelial cells. **b** Representative images of HE staining from 4-week mice. Black arrows: vessels. **c** Representative images of IF staining for CD31 from 4-week mice. White arrows: vessels. **d** Statistical analysis of vascular area (*n* = 4) in (**c**). **e** Details of blood vessels in trabecula (upper) and endosteum (lower) after tissue clearing. White arrows: vessels. **f** Representative IF staining for CD31 on drill-hole sections. White arrows: the vessels adjacent to tdTomato^+^ cells. **g** Statistical analysis of vascular area (*n* = 7) in (**f**). The scale bar in (**a**) is 20 μm; and in (**b**), (**c**), and (**f**) is 100 μm; and in (**e**) is 500 μm. GP growth plate, CB cortical bone, BM bone marrow

To confirm the role of PADs in angiogenesis, we analyzed femoral vasculature of *Col10a1-Cre; R26*^*DTA/+*^ and control mice. Immunostaining results using anti-CD31 antibody showed a close association between tdTomato^+^ cells and CD31^+^ endothelial cells in the endosteum, cortical bone, and marrow regions, and proliferating CD31^+^ cells in contact with tdTomato^+^ cells in *Col10a1-Cre; R26*^*tdT/+*^ mice (Fig. 4a). Unlike *Col10a1-Cre* mice, where numerous blood vessels accompanied trabecular bone, *Col10a1-Cre; R26*^*DTA/+*^ mice showed thicker trabeculae lacking comparable vascular ingrowth (Fig. 4b). In sharp contrast to *Col10a1-Cre; R26*^*tdT/+*^ mice, metaphyseal and cortical vascularization was apparently impaired in *Col10a1-Cre; R26*^*DTA/+*^ mice. In the diaphysis, where tdTomato^+^ cells were scarce or absent, vascularization remained comparable between the two mouse lines (Fig. 4c, d). To better visualize three-dimensional (3D) changes in vasculature, we employed tissue-clearing techniques and found that metaphysis was densely invaded by blood vessels in *Col10a1-Cre; R26*^*tdT/+*^ mice, whereas metaphysis *Col10a1-Cre; R26*^*tdT/DTA*^ mice exhibited poor vascular ingrowth (Fig. 4e, Fig. S5b, see supplementary video). Moreover, the number of blood vessels in the endosteal region and those crossing the cortical bone was markedly diminished (Fig. 4e). Next, we evaluated whether angiogenesis was impaired by PADs ablation during bone injury repair. In *Col10a1-Cre; R26*^*tdT/+*^ mice receiving the drilling injury, robust vascular ingrowth was observed at the injured region (Fig. 4f). However, area with vascular ingrowth was markedly decreased in *Col10a1-Cre; R26*^*DTA/+*^ mice post injury, as compared to that of controls (Fig. 4f, g). Together, these findings clearly demonstrated a role of PADs in metaphyseal and cortical vessel formation and post-injury angiogenesis.

### PADs promoted angiogenesis by secreting THBS4

**Fig. 5:**
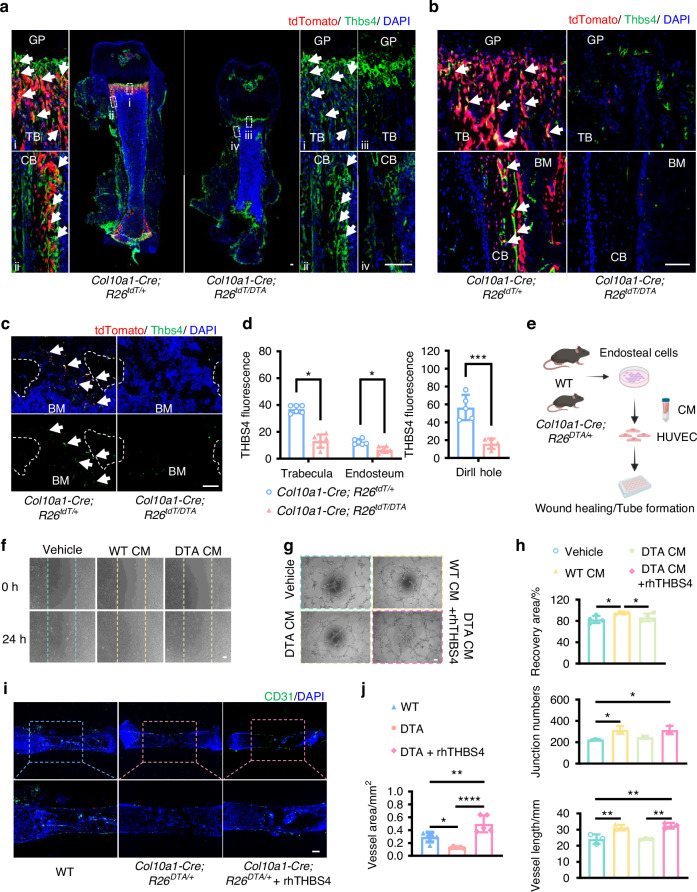
Descendants of HCs promoted angiogenesis through THBS4. **a**–**c** Representative IF images of THBS4 staining in P10 (**a**), 4-week (**b**), and drill-hole injury (**c**) tissues. (i-iv) Higher-magnification images. White arrows: the tdTomato^+^ cells expressing THBS4. **d** Statistical analysis of THBS4 staining fluorescence intensity in (**b**) (*n* = 6) and (**c**) (*n* = 5). **e** Experimental setup of testing CM from endosteal cells. **f** The effect of CM on the wound healing ability of HUVECs was evaluated by scratch experiment. **g** Representative images of tube formation assay with HUVECs. **h** Statistical analysis of scratch recovery area (*n* = 4) in (**f**) and quantification of tube formation (*n* = 3) in (**g**). **i** Representative images of IF staining for CD31 in metatarsals explants sections treated with or without rhTHBS4. **j** Statistical analysis of vascular area (*n* = 5) in (**i**). The scale bar in (**a**), (**b**), (**c**), (**f**), (**g**), and (**i**) is 100 μm. GP growth plate, TB trabecular bone, CB cortical bone, BM bone marrow, CM conditional medium, WT wild type, DTA, *Col10a1-Cre; R26*^*DTA*/+^

To confirm if THBS4 expression was reduced by depletion of PADs, we conducted THBS4 staining on femur tissues from *Col10a1-Cre; R26*^*tdT/+*^ mice and *Col10a1-Cre; R26*^*tdT/DTA*^ mice (Fig. 5a, b). The results showed that a subset of tdTomato^+^ cells co-stained with THBS4 in the trabecular bone and endosteal region of *Col10a1-Cre; R26*^*tdT/+*^ mice. Conversely, we found a significant reduction in THBS4 expression in these areas of *Col10a1-Cre; R26*^*DTA/+*^ mice (Fig. 5a, b, d). Moreover, expression of THBS4 was also significantly reduced in the injured region of *Col10a1-Cre; R26*^*tdT/DTA*^ mice receiving the drilling injury, as compared to that of *Col10a1-Cre; R26*^*tdT/+*^ controls (Fig. 5c, d). To evaluate THBS4 angiogenic impact, we isolated endosteal cells from wild-type and *Col10a1-Cre; R26*^*DTA/+*^ mice, then cultured HUVECs in medium conditioned by these cells (Fig. 5e). The medium conditioned with wild-type endosteal cells facilitated complete wound healing, which was not seen in the vehicle group or with the DTA medium after 24 h of co-culture (Fig. 5f, h). Moreover, conditioned medium from wild-type endosteal cells, or the addition of rhTHBS4 to the medium from *Col10a1-Cre; R26*^*DTA/+*^ cells, significantly enhanced angiogenesis, increasing both vascular junction formations and vessel length (Fig. 5g, h). In contrast, the medium from *Col10a1-Cre; R26*^*DTA/+*^ cells alone failed to promote angiogenesis (Fig. 5g, h). CCK8 assays revealed that rhTHBS4 enhanced proliferation of HUVECs in a dosage-dependent manner (Fig. S6a). More importantly, supplying THBS4 was sufficient to ameliorate the defective angiogenesis of *Col10a1-Cre; R26*^*DTA/+*^ metatarsal explants (Fig. 5i, j). Collectively, these data support THBS4 as a major secreting factor mediating pro-angiogenic effects of PADs.

## Discussion

Building on our previous work,^5,10^ the present study further elucidated that the lineage extension of HCs promoted the linear growth of long bones, contributed to the formation of trabecular bone, and facilitated the repair of bone injuries. Additionally, we utilized single-cell sequencing to uncover a new subpopulation of HC descendants, PADs, which was the most profoundly affected cell cluster by genetic depletion of HC descendants. Furthermore, we showed that PADs were spatially associated with endosteal and cortical vasculature, and depletion of PADs in mice impaired vascularization and post-injury angiogenesis of bone. Ultimately, functional assays confirmed that the angiogenic factor, THBS4, mediated cell-cell communication between PADs and endothelial cells. In brief, these data highlighted an essential role of HC descendants in the regulation of bone elongation and injury repair by enhancing angiogenesis via THBS4 secretion.

Prior to the present study, several mouse lines have been established to delineate HC transformation, including *Col10a1-Cre* knockout mice (*Ctnnb1*, *Irx3*, *Irx5*, *Runx2*, and *Hgs*) and *Col2a1-Cre; R26*^*DTA/+*^ mice.^7–10,51^ However, most of the *Col10a1-Cre* knockout mice exhibited relatively minor skeletal phenotype characterized by a subtle loss of trabecular bone, and *Col2a1-Cre; R26*^*DTA/+*^ mice did not survive postnatal stage. A more faithful mouse line is, therefore, required to convince investigators of the pathophysiological implications of HC transformation. *Col10a1-Cre; R26*^*DTA/+*^mouse line constructed in the present study provides a valuable model by presenting non-prenatal lethality and striking skeletal phenotypes. Compared to reported *Col10a1-Cre* knockout mice, *Col10a1-Cre; R26*^*DTA/+*^ mice showed more convincing malformation of trabeculae and shortening of limb, for genetic depletion of cells could avoid compensation at molecular level. Intrigued by single-cell sequencing results, we found the loss of skeletal blood vessels in *Col10a1-Cre; R26*^*DTA/+*^ mice, which was also seen in *Col2a1-Cre; R26*^*DTA/+*^ mice.^51^ Interestingly, *Col2a1* lineage cells were reported to contribute to CD31^+^ blood vessels in both the calvarial and long bone,^51^ while we found no lineage extension of HC descendants to endothelia. Given that *Col2a1-Cre* labels much more cell lineages than *Col10a1-Cre*, it could be presumed that other non-HC-derived cells might contribute to endothelia. Moreover, the embryonic ablation of *Col2a1* lineage cells resulted in almost complete loss of long bone, whereas ablation of *Col10a1* lineage cells did not induce complete loss of metaphyseal bone where HC transformation was active. It is possibly because other lineage cells could partially compensate for the bone loss, albeit the structural abnormality.

It is notable that the unexpected expansion of hypertrophic zone was observed in *Col10a1-Cre; R26*^*DTA/+*^ mice. Based on increased TUNEL-positive HCs and decreased number of osteoclasts within the expanded zone, it could be explained by remnants of dead HCs and cartilage matrix upon deficits in clearance by osteoclasts. However, we cannot rule out the artificial contribution of DTA, for it deletes cells by inhibiting protein synthesis,^28,52^ which might entail cellular stress responses before cell death. In concert with this, we previously showed that the integrated stress response by misfolded protein was associated with the expansion of the hypertrophic zone seen in a transgenic mouse model, carrying a 13 bp deletion in *Col10a1*.^53^ Taken together, research addressing the growth plate changes in *Col10a1-Cre; R26*^*DTA/+*^ mice requires in-depth investigation of potential artifacts from DTA (e.g., cellular stress responses) and comparative evaluation of alternative tools for genetic cell lineage depletion. Notably, accumulating evidence has identified THBS4 as a stress-induced factor, warranting further investigation into its mechanistic roles.^54–56^ However, we observed an overall decrease of THBS4 in bones of *Col10a1-Cre; R26*^*DTA/+*^ mice, which is not directionally concordant, for stress response would have induced THBS4 expression. Moreover, DTA did significantly decrease the number of THBS4-secreting HC daughter cells and induce a striking vasculature phenotype. These findings validate *Col10a1-Cre; R26*^*DTA/+*^ mice as a faithful model to confirm a role of HC descendants in regulation of bone vasculature.

Combining single-cell sequencing and functional assays, we demonstrated for the first time that HC descendants regulate angiogenesis by secreting THBS4. Lines of evidence supported THBS4 as a potent angiogenic factors. Studies revealed that overexpression of *Thbs4* enhanced tumor angiogenesis, postnatal retinal vasculature development, and angiogenesis during skin wound healing, whereas *Thbs4-*deficient mice exhibited diminished angiogenesis and a transient decrease in articular cartilage thickness.^44,57,58^ In vitro assays, researchers documented the proangiogenic activity of THBS4 by promoting adhesion, migration and proliferation of vascular endothelia.^44,58^ Mechanistically, with Integrin α2 and gabapentin receptor α2δ-1 being its receptors,^44,59^ THBS4 has been reported to mediate angiogenesis in response to TGF-β1 and facilitate the PI3K/AKT pathway in endothelial cells.^43,58^ In concert with these studies, we found that supplying THBS4 was sufficient to promote proliferation and tube formation of endothelial cells and rescue defective angiogenesis of *Col10a1-Cre; R26*^*DTA/+*^ metatarsal explants.

While our findings highlight THBS4 as a potential mediator of angiogenic regulation in skeletal biology, the precise molecular mechanisms underlying its vascular-modulatory functions remain unresolved. Furthermore, although we identified multiple angiogenesis-associated factors (e.g., *Vegfa*, *Fn1*, *Cxcl1*, *Col6a1*, and *Col1a2*,) co-expressed by HC-derived populations, their hierarchical contributions to vascular niche formation and functional redundancy in skeletal development warrant systematic interrogation. Although we have demonstrated the critical role of THBS4 in vascular regulation in mice whether this protein exerts conserved biological functions in human vasculature requires further validation.

**Fig. 6:**
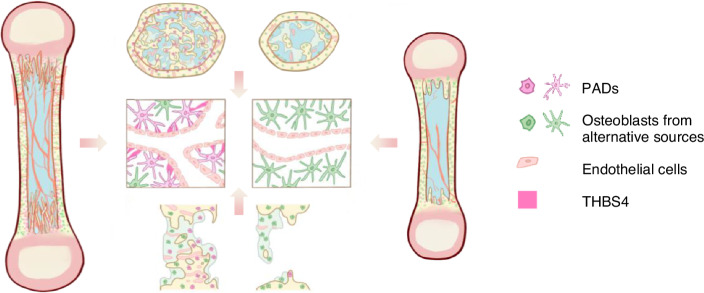
Schematic diagram of THBS4 secreted by PADs to regulate angiogenesis during bone growth and injury repair. PADs regulated angiogenesis by secreting THBS4 during bone growth and injury repair, but the removal of HCs and PADs hindered vessel growth, resulting in stunted growth and delayed repair

Collectively, the present study demonstrates a critical role of HC descendants in bone growth and injury repair by secreting THBS4 to regulate angiogenesis (Fig. 6). These findings also shed translational insights that could be leveraged to enhance injury repair of bone and treat defective angiogenesis.

## Methods and materials

### Animals

The *Col10a1-Cre* mice were generously provided by Professor Kathryn S. E. Cheah from the School of Biomedical Sciences at the University of Hong Kong.^5^ The *ROSA-DTA* mice were kindly gifted by Professor Zhou Bo from the Center for Excellence in Molecular Cell Science, Chinese Academy of Sciences (Jackson Laboratory, 010527). The *ROSA-tdTomato* mouse strain was purchased from the Jackson Laboratory (Bar Harbor, 007914). To trace HCs in mice’s long bones, we bred *Col10a1-Cre* mice with *ROSA-tdTomato* mice to obtain *Col10a1-Cre; R26*^*tdT/+*^ mice. We bred *Col10a1-Cre* mice with *ROSA-DTA* mice to obtain *Col10a1-Cre; R26*^*DTA/+*^ ablating HCs and their derived cells to investigate the pathophysiological significance of the transformation in long bone development. To test the efficiency of HC ablation, we bred *Col10a1-Cre; R26*^*tdT/+*^ mice with *ROSA-DTA* mice to obtain *Col10a1-Cre; R26*^*tdT/DTA*^ mice. The study utilized C57BL/6 mice housed in a Specific pathogen-free facility at the Air Force Medical University’s Experimental Animal Center. Mice were anesthetized and euthanized via cervical dislocation. All procedures adhered to the “Ethical Norms for Experimental Animal Welfare” guidelines of the Air Force Medical University.

### Histological analysis

For histological analysis, mice femurs were fixed in 4% paraformaldehyde overnight. Decalcification was performed using 10% EDTA for 2 weeks. Decalcified tissues were embedded in OCT compound (Leica) and cryosectioned at 7 μm using a cryostat (Leica). Cryosections were stained with HE, Safranin O/Fast Green (Solaribia, G1371), Goldner (Servicebio, 1064) and TRAP (Sigma, 386 A) as previously described.^60–62^

### Immunofluorescence staining and TUNEL assay

Immunofluorescence was performed as described previously.^10,62^ The primary antibodies used were anti-COL X (Abclonal, A18604, 1:200 dilution), anti-SOX9 (Abcam, ab185966, 1:100 dilution), anti-SP7 (Abcam, ab209484, 1:200 dilution), anti-CD31 (R&D Systems, AF3628, 1:150 dilution), anti-Ki67 (Invitrogen,14-5698-82, 1:200 dilution) and anti-THBS4 (Santa Cruz, sc-390734, 1:100 dilution). The primary antibodies were detected using species-specific Alexa Fluor-conjugated secondary antibodies (Abcam). Apoptotic cells in femur cryosections were identified using the TUNEL assay (Beyotime, C1091) as per the kit’s guidelines. All sections were mounted with ProLong Gold Antifade Mountant with DAPI (Invitrogen) and visualized using a fluorescent microscope (ZEISS or OLYMPUS). The number of positive cells was counted using Adobe Photoshop 2020. Quantification of the vessel area and fluorescence intensity were measured with ImageJ.

### Drill hole surgery

Drill-hole surgery was mentioned before.^16^ Mice were anesthetized with 1% pentobarbital sodium via intraperitoneal injection (10 µL/g). For drill-hole injuries, the femurs of 8-week-old (8-week) *Col10a1-Cre; R26*^*tdT/+*^ *and Col10a1-Cre; R26*^*DTA/+*^ mice were surgically prepared. A cut was made in the lateral skin close to the femur’s distal end. The muscle was split, and the periosteum was peeled back to reveal the femoral surface. A 0.5 mm diameter hole was drilled into the distal end of the femurs using a Microdrill (RWD Life Science, 87001).

### Micro-CT analysis

Micro-CT analysis was performed to evaluate bone microstructure, as previously reported.^62^ Briefly, femurs were collected and scanned using the following settings: 8 µm isotropic voxel size, 48 kV peak tube voltage, and 200 µA current (SkyScan 1276, Bruker). Three-dimensional images were reconstructed and regions of interest analyzed using NReconServer, CTAn, and CTvox software (GE Health Care Co.) as per the manufacturer’s guidelines. Trabecular bone parameters in the distal metaphysis were quantified using 150 histological sections from *Col10a1-Cre; R26*^*tdT/+*^ mice and 120 sections from *Col10a1-Cre; R26*^*tdT/DTA*^ mice, with the number of sections per bone adjusted proportionally to individual bone length. Cortical bone parameters were assessed with 50 slices, 1 mm below the trabecular area. Drill-hole bone parameters were evaluated using 160 slices from the drill-hole region.

### Three-point bending test

Fresh, unfixed tibias were tested using a 3220 ElectroForce Tester (ELF3220, TA Instruments) to evaluate their structural and material strength in a three-point bending test. A constant loading rate of 0.02 mm/s was applied to the tibial midshaft, between two supports that were 8.0 mm apart, with load-deformation curves recorded until fracture. The maximum load at failure and Elastic modulus (MPa) were calculated as previously reported.^62,63^

### Single-cell sequencing cell preparation and sequencing

*Col10a1-Cre; R26*^*tdT/+*^ (*n* = 3) and *Col10a1-Cre; R26*^*tdT/DTA*^ (*n* = 2) mice, aged 4 weeks (4-week), were sacrificed. Tibias and femurs were dissected and subjected to serial digestions after removing soft tissues, as previously described.^32,64^ Briefly, bones were digested in 0.2% collagenase type I (Sangon, A004194) for 15 min at 37 °C to remove periosteal cells. Epiphyseal ends were excised and bone marrow was flushed out. Bone shafts were cut into 1–2 mm pieces and placed in collagenase type I for digestion, repeated 10 times. The collagenase fractions were then collected and centrifuged to harvest cells. Cell pellets were resuspended, filtered through a 70 µm strainer, and sent for sequencing on the NovaSeq platform.

### Integrative analysis of single-cell sequencing datasets

Unique molecular identifier (UMI) counts were obtained by aligning FASTQ files to the mouse reference GRCm38 through Cell Ranger (V.4.0) software. The single-cell transcriptome sequencing data of endothelial cells were downloaded from the GEO database (GSE128423). We performed integrative analyses following the Seurat pipeline (https://satijalab.org/seurat/articles/integration_introduction.html). The identified chondro-osteogenic and endothelial clusters were categorized into subsets for cell-cell communication. CellChat (version 1.4.0) was utilized to detect and decipher cell-to-cell communication within chondro-osteogenic and endothelial clusters. We adhered to the standard workflow for subsequent analyses, employing the default settings.^49^ Human and mouse single-cell datasets were integrated using Seurat’s MapQuery workflow.^65^ PADs were annotated with reference databases (GSE143753),^50^ assigning cells based on maximal prediction confidence scores.

### Tissue optical clearing technology

For 3D imaging, a significant challenge was to preserve the inherent tdTomato fluorescence during the tissue-clearing process. To overcome this, we utilized enhanced three-dimensional imaging of solvent-cleared organs with a superior fluorescence preserving capability (FDISCO) clearing method that successfully preserved tdTomato fluorescence during the tissue-clearing process, as described previously.^66^ In short, one hour before euthanizing the mice, 100 µL of Alexa Fluor 647 anti-mouse CD31 antibody (BioLegend, 102416, 25 µg/100 µL) was injected into the tail veins of both 4-week *Col10a1-Cre; R26*^*tdT/+*^ and *Col10a1-Cre; R26*^*tdT/DTA*^ mice to label blood vessels. Mice were perfused with 4% paraformaldehyde in PBS, and their femurs were then collected, fixed, and decalcified. The femurs underwent tissue-clearing using the FDISCO reagent kit (Jarvis, JA11012). Mouse femurs appeared transparent macroscopically after processing (Fig. S5a) and then were scanned with a light-sheet scanner (LiToneXL, Light Innovation Techno). The raw TIFF data from the scan was stitched and converted using LitScan software, after which it was imported into Imaris software for visualization.

### Endosteal primary cell culture

Single-cell sequencing digestion methods were used to isolate bone endosteal cells from postnatal day 10 (P10) wide type and *Col10a1-Cre; R26*^*DTA/+*^ littermate mice.^32,64^ The bone fragments were co-cultured with the digested cells, allowing more bone endosteal stem cells to emerge from the bone debris.^64^ Bone endosteal cells were cultured in MEM α medium (HyClone, SH30265.01) supplemented with 15% FBS. The medium was harvested and centrifuged using concentration tube (Millipore Amicon™, UFC801008D) at 4 000 × *g* for about 10 min.^67,68^ Following concentration, the conditioned medium was divided into aliquots and stored at –80 °C.

### Cell Counting Kit-8(CCK8) assay

HUVECs were cultured in ECM (Sciencell, 1001) supplemented with 15% FBS. For the cell proliferation assay, HUVECs were plated in 96-well plates at proper density and treated with rhTHBS4 recombinant protein (R&D, 2390-TH) at 0, 10, and 100 ng/mL concentrations, respectively, concentration previously validated by preliminary studies.^43,44^ CCK-8 reagent was cultured for 2 h after 0, 1, and 2 of incubation and then cell viability was measured at 450 nm wavelength.

### Wound healing assay

HUVECs were starved for an hour before digestion and then were seeded into a 24-well plate at 50 000 cells per well. HUVECs were subjected to a scratch wound assay using a yellow pipette tip after 2 days of culture in conditioned medium as described previously.^67,69^ After a gentle wash, cells were cultured in serum-free medium plus conditioned medium dilution from wide type and *Col10a1-Cre; R26*^*DTA/+*^ mice. The wound healing process was photographed at 24 h after injury. ImageJ software was utilized to evaluate the wound area.

### Tube formation assay

After 1 h serum starvation, HUVECs were digested and resuspended in a serum-free medium for seeding in 96-well plates precoated with Matrigel (Corning, 356234)^69^ at 30 000 cells per well. Conditioned media dilution from wide type, *Col10a1-Cre; R26*^*DTA/+*^, *Col10a1-Cre; R26*^*DTA/+*^ with equivalent 100 ng/L of rhTHBS4 solution were added to HUVECs. After four-hour incubation, tube branches of each well were photographed under the microscope. AngioTool software was used to quantify the number of junction and the total vessel length.

### Metatarsal culture

Metatarsal cultures were conducted as described previously.^60^ In brief, metatarsal explants were isolated from P7 mice of wide type and *Col10a1-Cre; R26*^*DTA/+*^ and cultured in 200 μL of BGJb medium (Gibco) supplemented with 0.2% w/v BSA, 5 μg/mL L-ascorbic acid phosphate, 1 mmol/L β-glycerophosphate, 0.05 mg/mL gentamicin and 1.25 μg Amphotericin B. To modulate rhTHBS4 in cultures, left metatarsals of *Col10a1-Cre; R26*^*DTA/+*^ were incubated in the medium containing 10 µg/mL of rhTHBS4, whereas the right counterparts were used as controls. After 2 weeks of culture, the samples were harvested for sectioning and staining.

### Data analysis

Data were presented as mean ± SEM. For data comparison, unpaired t-tests, multiple unpaired t-tests, and one-way ANOVA were employed as appropriate. Statistical analysis and graph creation were conducted using GraphPad Prism 10 software. *P < 0.05, **P < 0.01, ***P < 0.001, ****P < 0.000 1 indicated significance, while ns denoted no significance. All experiments included analysis of at least three independent experimental groups.

## Supplementary information

clean version_suppl_BONERES-04389R1

The video of the vasculature in the whole femur after clearing and imaging from *Col10a1-Cre; R26*^*tdT/+*^ mice

The video of the vasculature in the whole femur after clearing and imaging from *Col10a1-Cre; R26*^*tdT/DTA*^ mice

The video of the vasculature in the metaphysis after clearing and imaging from *Col10a1-Cre; R26*^*tdT/+*^ mice

The video of the vasculature in the metaphysis after clearing and imaging from *Col10a1-Cre; R26*^*tdT/DTA*^ mice
