# Supplementary material for: Descendants of hypertrophic chondrocytes promote angiogenesis by secreting THBS4 during bone growth and injury repair
Source: Bone Res. 2025 Nov 10;13:92. doi: 10.1038/s41413-025-00469-2 (PMC12598051; doi:10.1038/s41413-025-00469-2)
Supplement: Supplementary file 1 — clean version_suppl_BONERES-04389R1 [file 41413_2025_469_MOESM1_ESM.pdf]

Descendants of hypertrophic chondrocytes promote angiogenesis by secreting THBS4 during bone growth and injury repair

Shiju Song *et al*.

*Corresponding author. Email: yangliu@fmmu.edu.cn (Lead contact),

Chao Zheng: slc26a2@hotmail.com

This PDF file includes:

Figs. S1 to S6

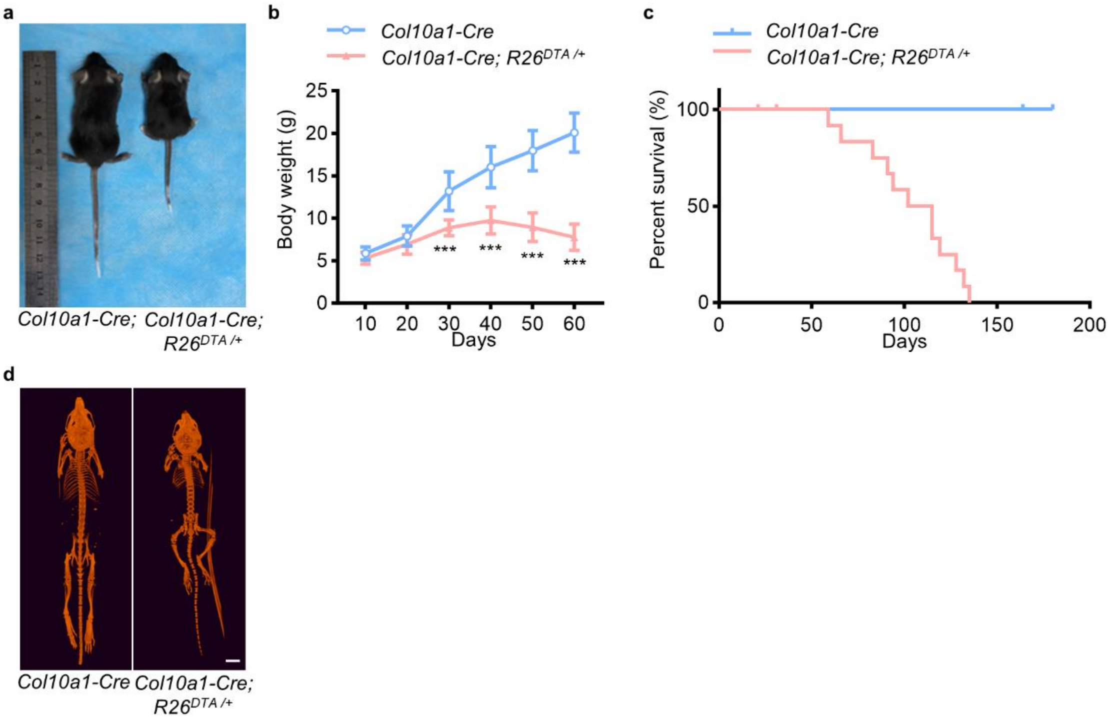

Fig. S1. Expression of DTA in HCs resulted in decreased body weight and shorter lifespan in mice. **a** Gross appearance from 4W mice. **b** Statistical analysis of body weight (n=12). **c** Kaplan-Meier survival curve (n=14). **d** Representative 3D reconstruction images of whole body from 4W mice. The scale bar in (d) is 5 mm.

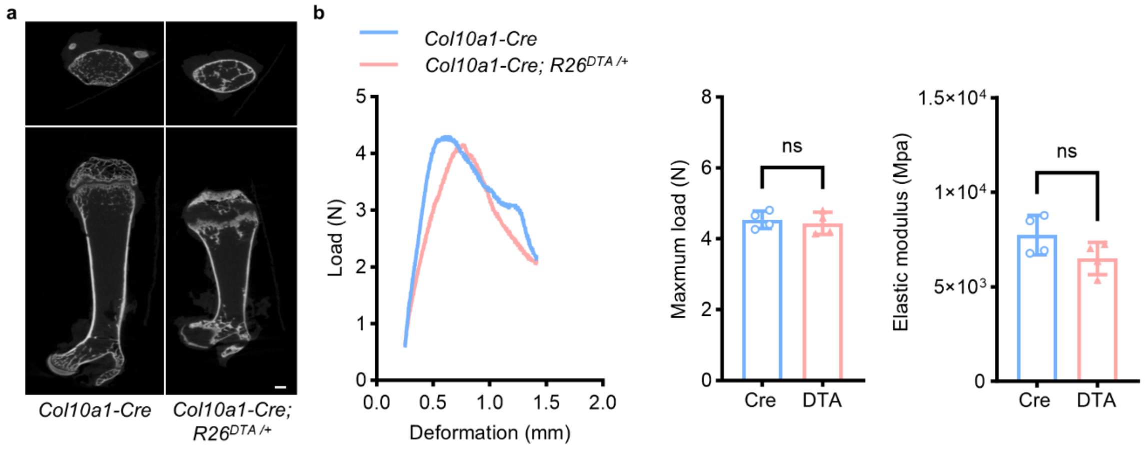

Fig. S2. Ablation of HCs did not impact mechanical function. **a** Representative image of the scanning section from 4W mice. **b** Representative load-deformation curve and statistical analysis of maximum load and elastic modulus (n=4) from three-point bending test of 4W mice. The scale bar in (a) is 500 µm.

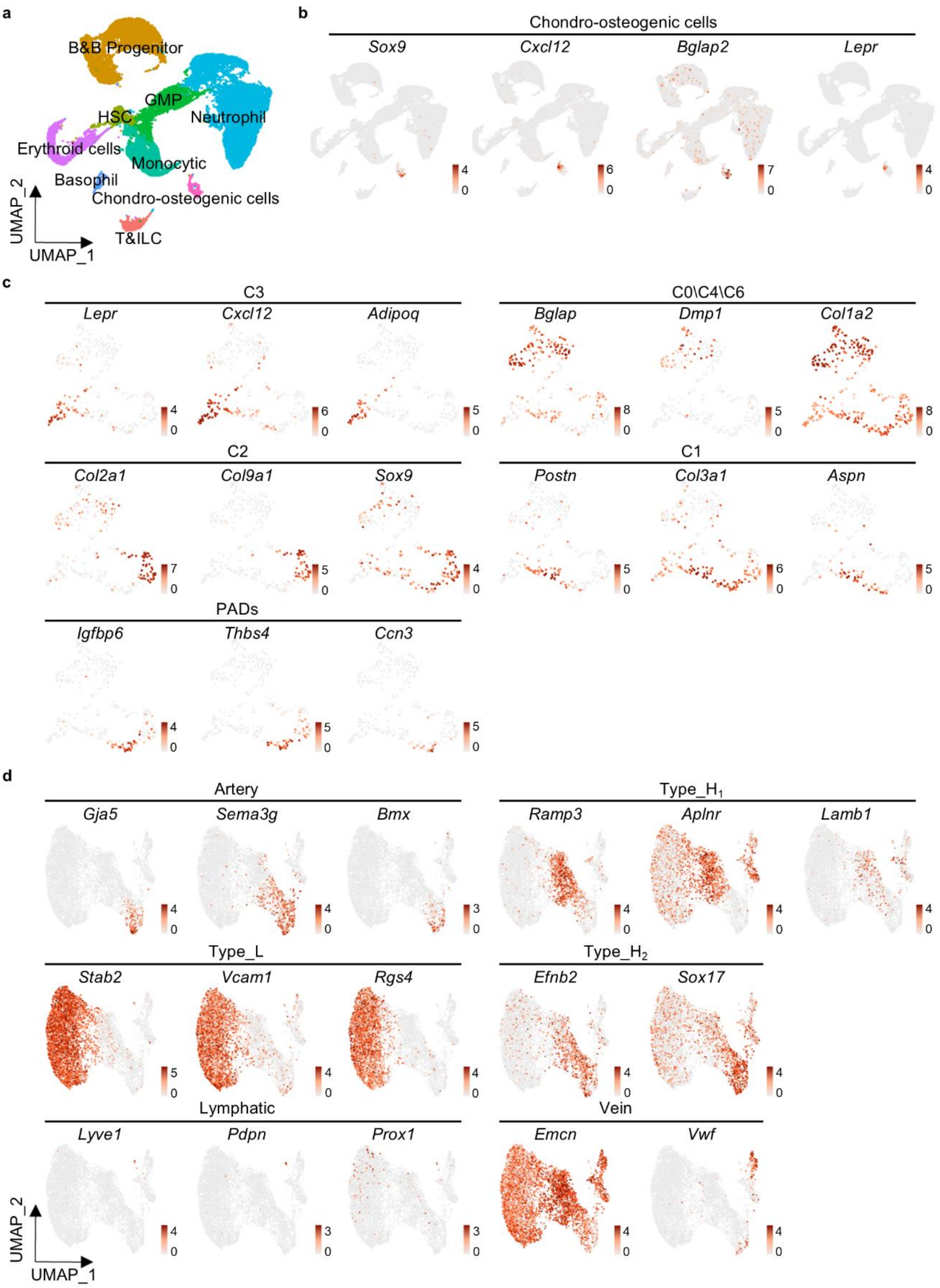

Fig. S3. Feature plots visualized marker genes of cells from the single-cell sequencing data. **a** UMAP visualization of major sub-clusters of all cells captured by single-cell sequencing. **b** Feature plots showed markers for subclusters of all cells. **c** Feature plots showed markers for

subclusters of chondro-osteogenic cells. **d** Feature plots showed markers for subclusters of endothelial cells. B&B Progenitor, Mature B cell and B Progenitor cell; GMP, Granulocyte-macrophage Progenitor; Neutrophil, Neutrophil Progenitor and Neutrophil; Monocytic, Monocyte, Macrophage and DC; HSC, Hematopoietic Stem Cell; T&ILC, Naïve T cell and Innate Lymphoid Cells.

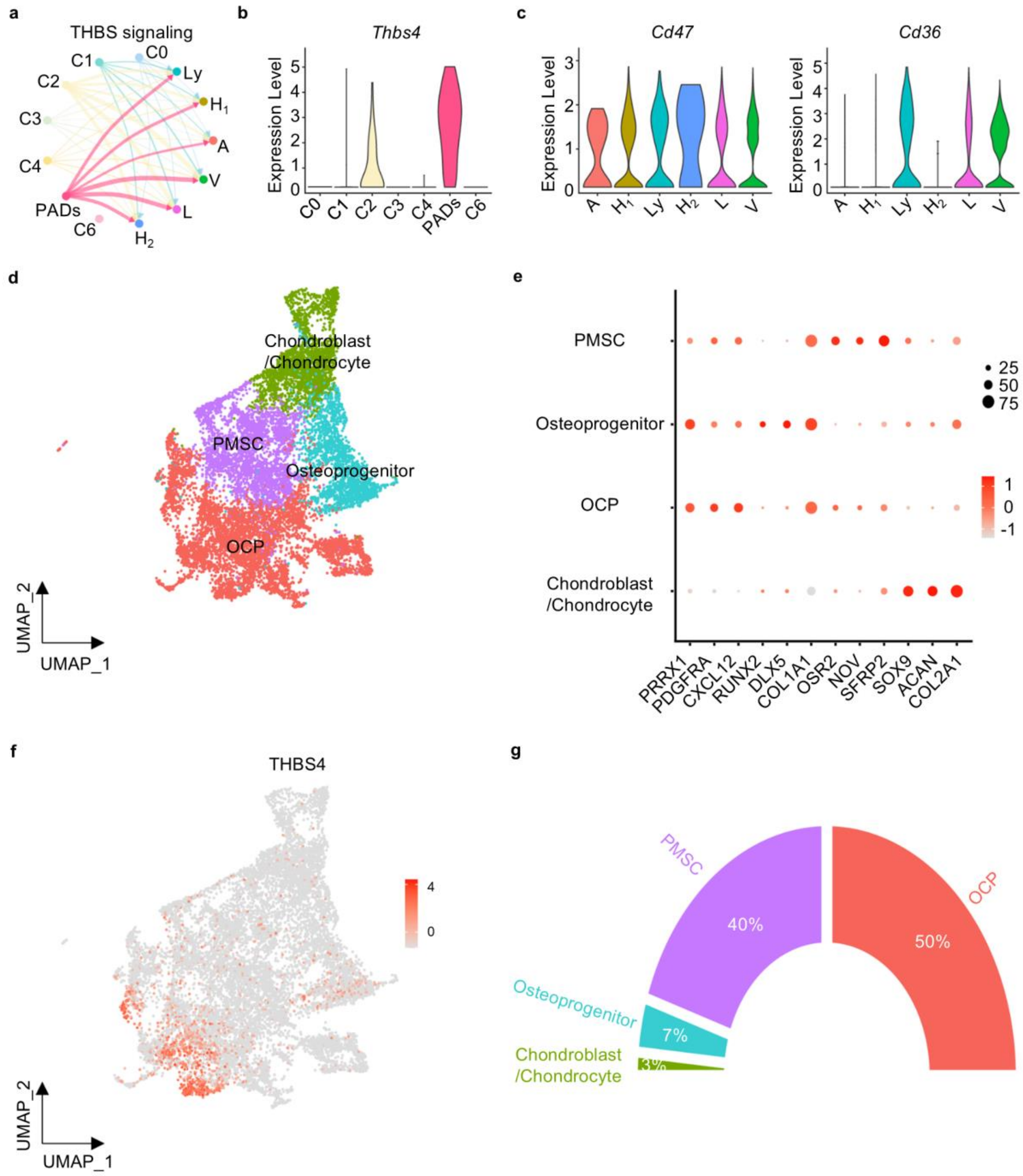

Fig. S4. Validation of THBS4 signaling between chondro-osteogenic progenitors and endothelial cells and THBS4 in human skeletal stem cell (SSC) niches. **a** CellChat interaction analysis of chondro-osteogenic cells and endothelial cells via the THBS signaling pathway. **b** Violin plots showed the relative expression of *Thbs4* in chondro-osteogenic cells. **c** Violin plots showed the relative expression of *Cd47* and *Cd36* in endothelial cells. **d** UMAP visualization of major SSC subtypes in the human dataset. **e** Feature plots highlighting canonical marker genes defining distinct SSC subpopulations. **f** Spatial expression pattern of THBS4 across the

UMAP. **g** Proportional composition analysis of mapped cell subtypes within PADs. OCP, Osteo-chondrogenic Progenitor; PMSC, Perichondrial Mesenchymal Stromal Cell.

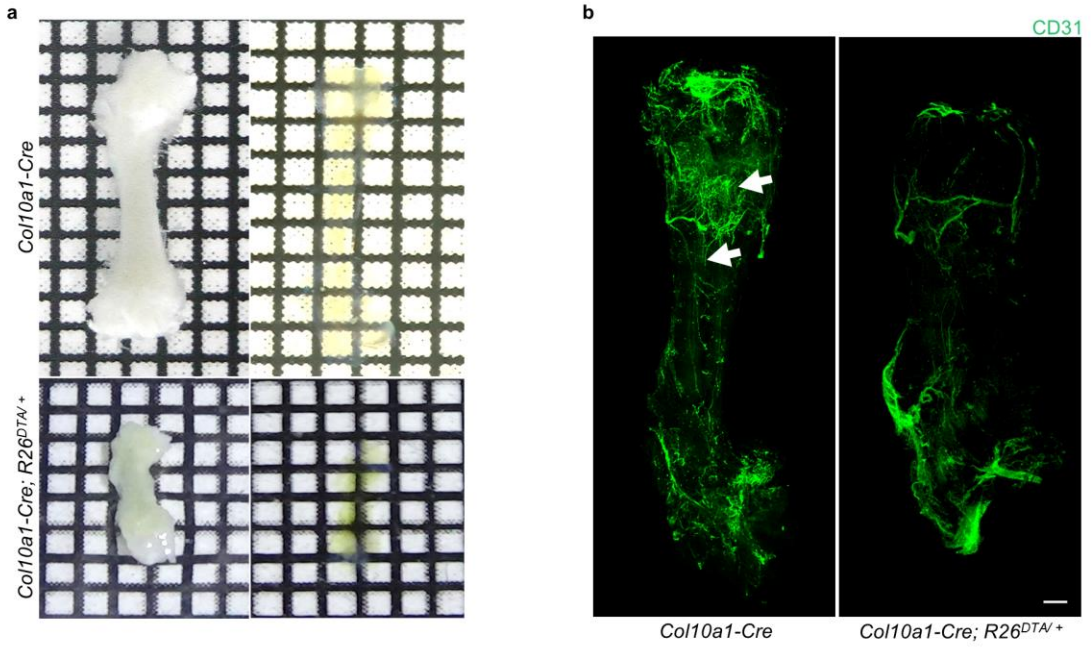

Fig. S5. Gross appearance before and after the tissue-clearing process. **a** Bright-field images of femurs from 4w mice before (left) and after (right) clearing. **b** 3D reconstruction of the vasculature in the whole femur after clearing and imaging from 4W mice. The scale bar in (b) is 500 μm.

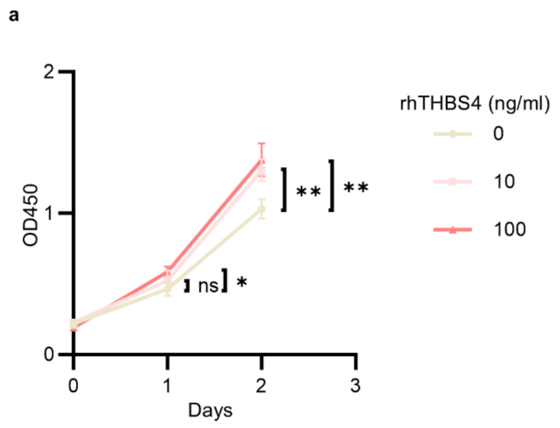

Fig.S6. The concentration of rhTHBS4 was explored using the CCK8 assay. HUVECs were exposed to varying concentrations of rhTHBS4 (0, 10, and 100 ng/ml). **a** CCK-8 quantitative results of HUVECs in different concentrations over the course of 2 days (n=4).
